# Derivation of Human Corneal Keratocytes from ReLEx SMILE Lenticules for Cell Therapy and Tissue Engineering

**DOI:** 10.3390/ijms24108828

**Published:** 2023-05-16

**Authors:** Maria A. Surovtseva, Irina I. Kim, Natalia A. Bondarenko, Alexander P. Lykov, Kristina Yu. Krasner, Elena V. Chepeleva, Nataliya P. Bgatova, Alexander N. Trunov, Valery V. Chernykh, Olga V. Poveshchenko

**Affiliations:** 1 Research Institute of Clinical and Experimental Lymphology-Branch of the Institute of Cytology and Genetics, Siberian Branch of Russian Academy of Sciences, 2 Timakova Str., 630060 Novosibirsk, Russia; 2 Novosibirsk Branch of S. Fedorov Eye Microsurgery Federal State Institution, 10 Kalkhidskaya Str., 630096 Novosibirsk, Russia

**Keywords:** corneal fibroblasts, keratocytes, reversion, proliferation, migration, cytokines

## Abstract

Fibroblasts isolated and expanded from ReLEx SMILE lenticules can be a source of human keratocytes. Since corneal keratocytes are quiescent cells, it is difficult to expand them in vitro in suitable numbers for clinical and experimental use. In the present study, this problem was solved by isolating and growing corneal fibroblasts (CFs) with a high proliferative potential and their reversion to keratocytes in a selective serum-free medium. Fibroblasts reversed into keratocytes (rCFs) had a dendritic morphology and ultrastructural signs of activation of protein synthesis and metabolism. The cultivation of CFs in a medium with 10% FCS and their reversion into keratocytes was not accompanied by the induction of myofibroblasts. After reversion, the cells spontaneously formed spheroids and expressed keratocan and lumican markers, but not mesenchymal ones. The rCFs had low proliferative and migratory activity, and their conditioned medium contained a low level of VEGF. CF reversion was not accompanied by a change with the levels of IGF-1, TNF-alpha, SDF-1a, and sICAM-1. In the present study, it has been demonstrated that fibroblasts from ReLEx SMILE lenticules reverse into keratocytes in serum-free KGM, maintaining the morphology and functional properties of primary keratocytes. These keratocytes have a potential for tissue engineering and cell therapy of various corneal pathologies.

## 1. Introduction

The human corneal stroma contains human corneal stromal cells (hCSCs). The term hCSCs is used for both corneal keratocytes and corneal fibroblasts. hCSCs in a healthy cornea are represented by mitotically resting immobile keratocytes. They perform the function of producing and maintaining the extracellular matrix, ensuring morphostructural and biochemical stability, and the transparency of the corneal tissue [1,2]. Keratocytes can transform into fibroblasts and myofibroblasts under the influence of TGF-β, IL-1 when thecornea is damaged. Corneal fibroblasts (CFs) are activated keratocytes, of which the pronounced proliferative activity is essential for rapid cell expansion. CFs act as signal cells by attracting inflammatory cells. They are involved in the restoration of the cornea, producing collagen, elastin, proteoglycans, and glycoproteins. The activation of keratocytes in fibroblasts is also observed in vitro when cultured in a medium supplemented with serum [3]. After the repair of corneal damage (in the absence of TGF-β, IL-1) or when cultured in a serum-free keratocyte growth medium (KGM) supplemented with ascorbic acid, ITS and bFGF, CFs can be reversed into resting keratocytes [4,5]. CFs can transform into myofibroblasts through an excessive stimulation of TGF-β. Myofibroblasts are involved in corneal regeneration by causing wound contraction, fibrous extracellular matrix organization, and scar formation [6,7]. They are terminated when the tissues are repaired by apoptosis or deactivated [6].

Fibrosis of the stroma during its damage (burn, traumatic, after surgical interventions) leads to blindness [8]. In addition, the pathogenesis of many ectatic conditions, as well as genetically determined anomalies and congenital dystrophies of the cornea, is associated with increased apoptosis and a loss of keratocytes [9]. Corneal blindness is the third leading cause of low vision after cataracts and glaucoma [10]. About 12.7 million people in the world are in need of donor cornea transplantation [11]. Due to the shortage of donor corneas, new approaches to the treatment of corneal blindness are being sought. Such approaches are cell therapy and the development of tissue-engineered corneal constructs, which are an alternative to corneal allotransplantation. The development of these approaches is associated with the availability of corneal stroma cells, i.e., keratocytes. The quality of the restoration of the cornea is determined by the phenotype of keratocytes in the transplant.

Earlier studies have shown that human cornea can be used as a source of hCSCs. For these purposes, a primary keratocyte culture is obtained from the cornea by peeling the epithelium and removing the corneal endothelium [12,13]. However, due to the shortage of donor corneas, hCSCs are usually isolated from the corneoscleral rings of the peripheral cornea removed during keratoplasty. Since keratocytes in the corneal stroma are distributed unevenly, about 10% of vimentin-positive keratocytes are located in the areas of Bowman’s and Descemet’s membranes, so some researchers consider it a necessary condition for an effective differentiation of keratocytes into fibroblasts and myofibroblasts in case of corneal damage [14]. The density of stromal cells decreases from the anterior to the posterior sections of stroma cornea [15]. In this regard, it would be advantageous to use the anterior–central corneal part as a source of primary hCSC culture.

Small incision lenticule extraction (ReLEx SMILE) is widely used to correct myopia and myopic astigmatism [16]. The decellularized corneal stromal equivalents obtained from the ReLEx SMILE lenticules are a novel and accessible source of biocompatibility collagen-rich extracellular matrix scaffolds for regenerative medicine [17]. ReLEx SMILE lenticules are a safe and effective alternative for treating corneal ulcers and perforations in animals [18] and humans [19]. Keratoplasty using SMILE lenticules is often performed for treatment of post-LASIK corneal ectasia [20], presbyopia [21], and in surgical treatment of keratoconus [22]. There are also two publications describing the CFs isolated from lenticules [23,24].

In recent years, the number of surgical corrections of myopia and astigmatism using ReLEx SMILE has significantly increased. Since most patients to have undergone ReLEx SMILE are conditionally healthy young people, many healthy corneal lenticules are obtained after the correction. Considering the deficiency of corneas, especially their anterior–central part, we propose using fresh lenticules as a source of hCSCs in vitro. The method is especially advantageous for lenticules, since it does not require removing the epithelial and endothelial cells from the outer and inner surfaces. Therefore, corneal stromal lenticules become an accessible source for isolating a ‘pure’ culture of corneal stromal cells.

The aim of this study was to derive keratocytes through the reversal of corneal fibroblasts from ReLEx SMILE lenticules, and study the morphofunctional changes accompanying the reversion of fibroblasts to keratocytes.

## 2. Results

### 2.1. Morphology of hCSCs

**Figure 1:**
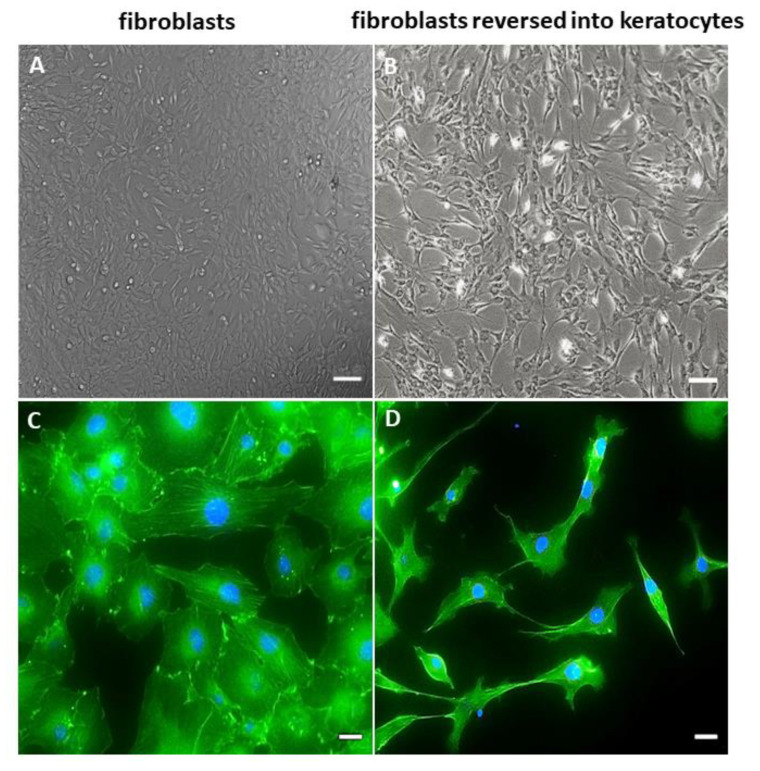
Morphology of the hCSCs derived from lenticules. (**A**) Phase–contrast micrographs of corneal fibroblasts derived from lenticules. (**B**) Phase–contrast micrographs of cells cultured for 21 days in KGM. The scale bar is 100 µm. F-actin (green, phalloidin) staining is counterstained with DAPI (blue). (**C**) Corneal fibroblasts. (**D**) Fibroblasts reversed into keratocytes. The scale bar is 20 µm.

The cultures grown in a DMEM/F12 medium supplemented with 10% FCS consisted of the cells adhered to a plastic surface. Cells were elongated, acquired a spindle shape, and formed colonies (Figure 1A). The fibroblast-like cell monolayer reached 80–90% confluence within 3–4 weeks. The staining of their cytoskeleton with phalloidin demonstrated the presence of the developed network of F-actin fibers with distinct bundles of intracellular actin filaments (Figure 1C).

Cultivation in selective serum-free KGM for 21 days changed CF morphology and phenotype, and reversed them into keratocytes. The rCFs acquired a dendritic shape and formed long cytoplasmic processes. They formed patterns shaped as waves and arcs (Figure 1B). The actin cytoskeleton stained with phalloidin demonstrated a sparse network of F-actin fibers with a predominantly cortical actin organization (Figure 1D).

### 2.2. Transmission Electron Microscopy of hCSCs

**Figure 2:**
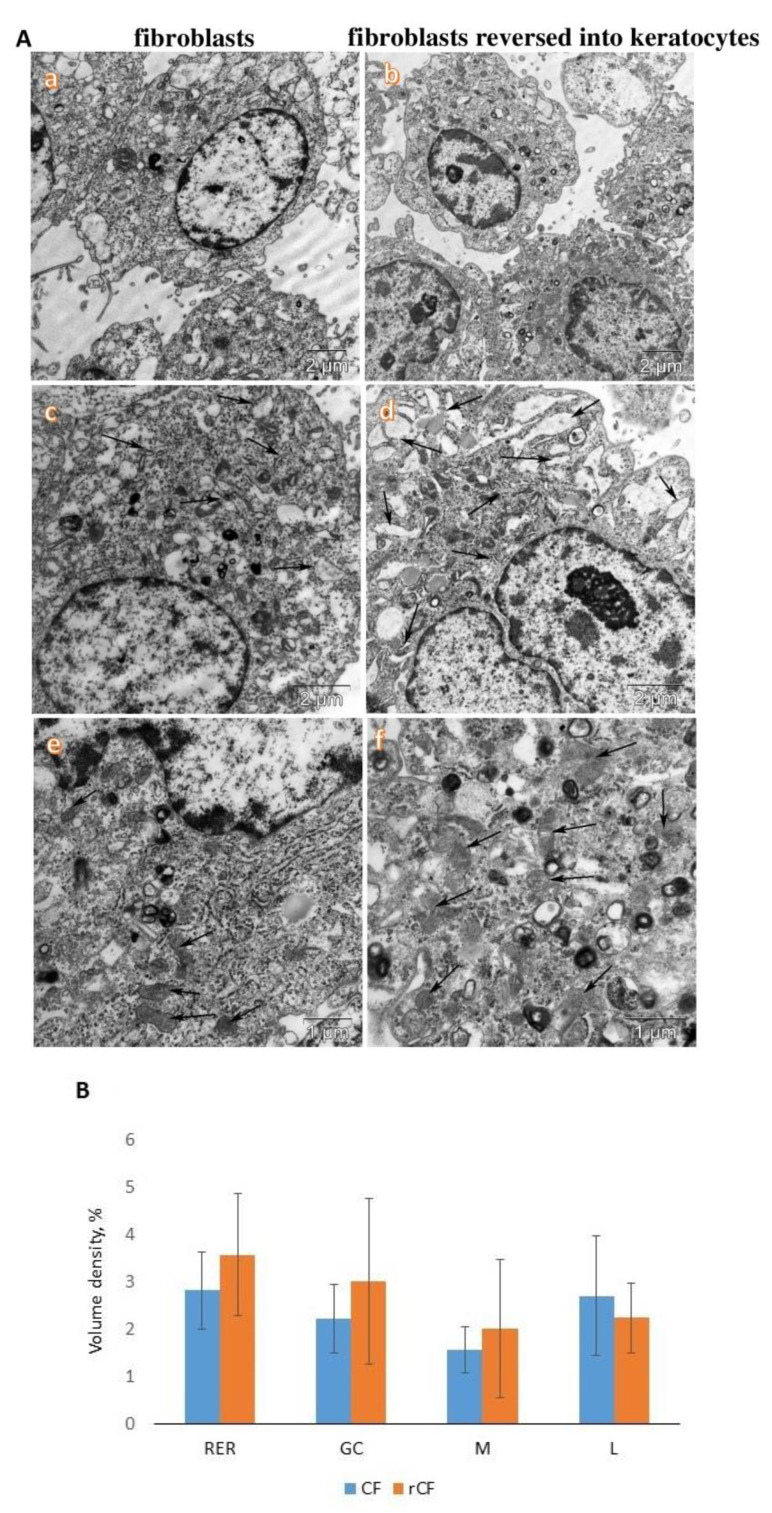
Ultrastructure of corneal fibroblasts (CFs) and fibroblasts reversed into keratocytes (rCF). (**A**) Transmission electron microscopy of cells. Lower electron density and organelle saturation of CF cells (**a**) compared to rCF cells (**b**). Different content of rough endoplasmic reticulum membranes (arrows) in CF cells (**c**) and rCF cells (**d**). Mitochondrial content (arrows) in CF cells (**e**) and rCF cells (**f**). (**B**) Volumetric density of membranes of the rough endoplasmic reticulum (RER), the Golgi complex (GC), mitochondria (M), and lysosomes (L).

We observed that rCFs had a higher electron density of the cytoplasm and content of organelles than CFs (Figure 2A,B). In particular, rCFs were characterized by a higher content of membranes of the rough endoplasmic reticulum than CFs. The cisterns of rCFs were often dilated (Figure 2A(c,d)). In addition, a higher content of mitochondria was observed in rCFs (Figure 2A(e,f)). Large activated nucleoli in the nucleus of rCFs were observed (Figure 2A(b,d)).

A comparative morphometric analysis of rCFs and CFs did not reveal significant differences in their ultrastructural organization. Only tendencies toward an increase in volumetric density of the membranes of the rough endoplasmic reticulum, the Golgi complex, and mitochondria were observed.

### 2.3. Phenotype of hCSCs

**Figure 3:**
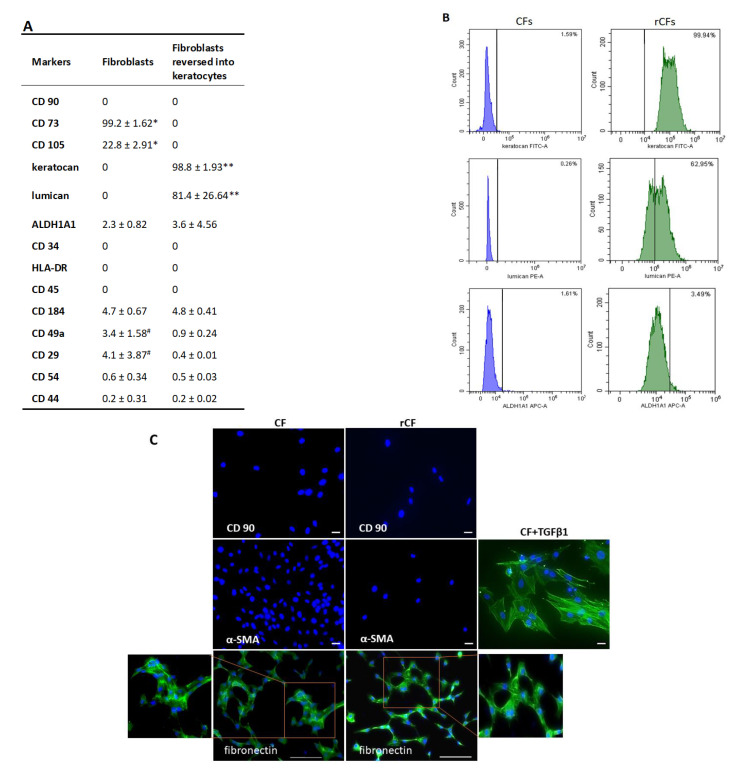
hCSC characteristics: (**A**) percentage of various fibroblast and keratocyte phenotype markers, adhesive molecule markers in two hCSC populations: CFs (*n* = 13), rCFs (*n* = 8); * *p* < 0.05, ** *p* = 0.003, # *p* = 0.01. (**B**) Percentage of CFs and rCFs that expressed keratocyte markers. (**C**) Fluorescence microscopy images showing the comparative expression of various markers in hCSCs: green—stained with fibronectin and α-smooth muscle α actin antibodies (induction of myofibroblasts with addition of TGFβ1, positive control), blue—stained with DAPI for nuclei. The scale bar is 100 µm for the fibronectin and 20 µm for the other markers.

The hCSCs cultured in the presence of 10% serum-expressed markers of mesenchymal cells, CD73 (99.2 ± 1.62%) and CD105 (22.8 ± 2.91%), were negative to CD90, CD34, CD45, HLA-DR, as well as to keratocan and lumican. Flow cytometric analysis showed that the CFs expressed poorly, such ALDH1A1, along with adhesive molecules, such as CD29, CD49a, and CD184 (Figure 3A). Immunocytochemistry also confirmed the absence of CD90 and alpha-SMA, but revealed the presence of fibronectin (Figure 3C).

After the reversion, the cells expressed high levels of keratocyte markers such as keratocan (98.8 ± 1.93%) and lumican (81.4 ± 26.64%) (Figure 3B). The expression of ALDH1A1 remained practically unchanged. Mesenchymal cell markers (CD90, CD73, and CD105) were absent. While the expression of adhesion molecules CD49a and CD29 reduced (*p* = 0.01), that of CD44, CD54, CD184 remained unchanged (Figure 3A). Immunocytochemistry showed the absence of CD90 and alpha-SMA, but revealed the presence of fibronectin (Figure 3C).

### 2.4. Spontaneous Sphere Formation

**Figure 4:**
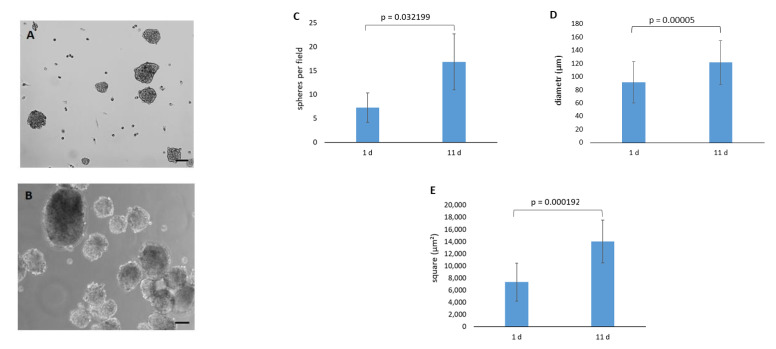
rCF spheres. (**A**) Phase–contrast micrographs of the spheres on day 1. (**B**) Phase–contrast micrographs of the spheres on day 11; scale bar = 100 µm. (**C**) Number of formed spheres. (**D**) Diameter of the spheres. (**E**) Area of the spheres. Data presented as the mean ± SD (*n* = 2).

The spontaneous formation of spheres was observed during rCF cultivation in the KGM medium after reaching confluence. Cells formed loose spheres of which the number, diameter, and area increased by day 11 (Figure 4C–E). The spheres did not attach to the plastic surface, increased in size, and floated for up to two weeks. They adhered to the plastic surface only after adding serum to the culture medium.

### 2.5. hCSC Proliferation

**Figure 5:**
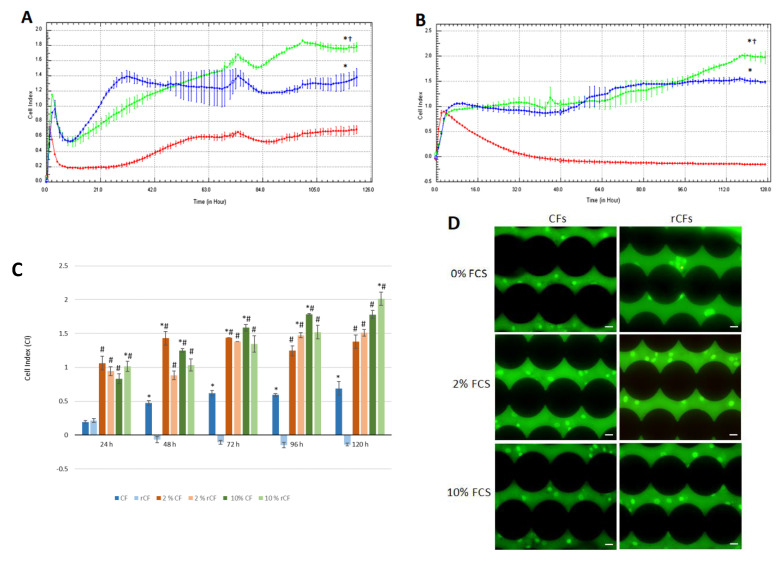
hCSC real-time proliferation traced by the xCELLigence system. (**A**) CF and (**B**) rCF proliferation. Red line—0% FCS, blue line—2% FCS, green line—10% FCS. Presented are an averaged experiment with triplicate determinations ± SD. * *p* < 0.05 in comparison with the control (0% FCS), † *p* < 0.05 in comparison with 2% FCS. (C) Histogram shows CI for the compared CF and rCF proliferations. c CF—control of CF (0% FCS), c rCF—control of rCF (0% FCS). Presented are the mean values of different experiments ± SD (*n* = 3). ^#^ *p* < 0.05 compared to the corresponding control (0% FCS), * *p* < 0.05 indicates differences between the CF and rCF. (D) hCSC fluorescent staining was performed with acridine orange (green, live cells) and propidium iodide (yellow, apoptotic cells at the end of the experiment, the black circles are electronic sensors located at the bottom of the wells. The scale bar is 20 µm.

To estimate hCSC proliferation, the CIs of CFs and rCFs for different (0, 2 and 10%) FCS concentrations were considered. For the first 3 h, CF CI for 10% FCS (CI = 1.1 ± 0.02) was higher than those for control (0% FSC; CI = 0.56 ± 0.01) and 2% FCS (CI = 0.8 ± 0.1) (*p* < 0.05), which was due to cell adhesion. From 4 to 15 h into the experiment, CI decreased for both 2% and 10% FCS and was similar, but higher than that for the control (*p* < 0.05), which was due to CF spreading. Then, the CI for 2% FCS sharply rose to 1.4 ± 0.5 and remained stable until the end of the experiment. From 70 to 120 h into the experiment, CF proliferation for 10% FCS (CI = 1.5 ± 0.04) was significantly higher than those for 2% FCS (CI = 1.28 ± 0.01) and the control (CI = 0.62 ± 0.04) (*p* < 0.05), of which the CI remained low until the end of the experiment (0.68 ± 0.04; see Figure 5A).

The CI of rCFs for 0%, 2%, and 10% FCS remained similar (≈CI = 0.9) during the first 4 h of the experiment, which was due to cell attachment and adhesion. Starting the fifth hour, the CI for 0% FCS began to decline and reached a plateau (CI = 0.17 ± 0.06). After 48 h, the CI for 2% FCS increased and remained higher than that of the control (0% FSC) until the end of the experiment (*p* < 0.05). The rCF CI for 10% FCS increased after 64 h (CI = 1.1 ± 0.08) and reached a plateau. At the end of the experiment, the CI for 10% FCS was significantly higher than that for 2% FCS (CI = 2 ± 0.05 and CI = 1.5 ± 0.05, respectively) and the control (*p* < 0.05) (Figure 5B).

Figure 5C shows that the rCF CIs at 24 h and 120 h for 10% FCS were significantly higher compared to those of fibroblasts (*p* < 0.05), but decreased significantly at other time points (*p* < 0.05). The rCF CI of the control sharply decreased after 24 h and remained at a low level until the end of the experiment. The CF CIs for 2% FCS (48 h and 72 h) and 10% FCS (48 h, 72 h and 96 h) were higher compared to those of rCFs (*p* < 0.05). By the end of the experiment, the CF and rCF CIs were similar for 2% FCS. However, for 10% FCS, the rCF CI was significantly higher than that of the CFs (Figure 5C).

Fluorescence microscopy images in Figure 5D show that in the control (0% FCS), the rCFs remained alive after 120 h of the experiment, despite the fact that they neither proliferate nor migrate (green, live cells).

### 2.6. hCSC Migration

**Figure 6:**
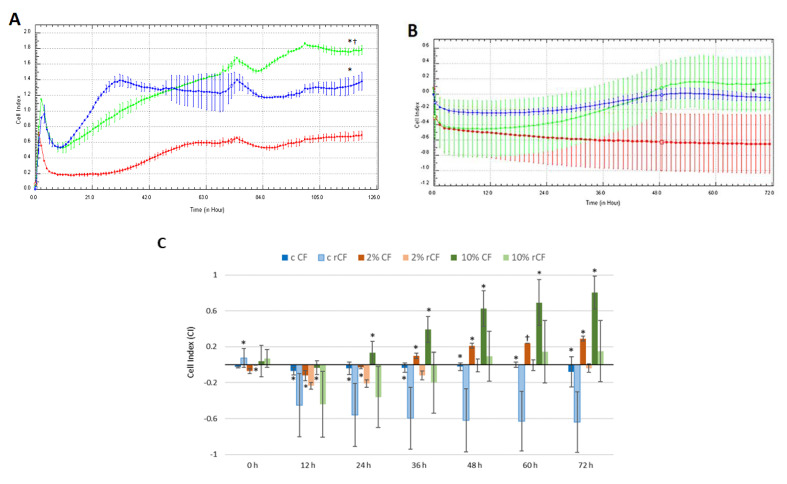
hCSC migration in real-time traced by the xCELLigence system. (**A**) CF and (**B**) rCF migration. Red line—0% FCS, blue line—2% FCS, green line—10% FCS. * *p* < 0.05 in comparison with the control (0% FCS), † *p* < 0.05 in comparison with 2% FCS. (**C**) Histogram shows the CIs of compared CF and rCF migration patterns. c CF—control of CF (0% FCS), c rCF—control of rCF (0% FCS). Presented are the mean values of different experiments ± SD (*n* = 3). * *p* < 0.03 and † *p* = 0.02 indicate differences between the CF and rCF.

It was found that CFs did not migrate in the 0% FCS medium (Figure 6A). They were at quiescent phase and practically did not migrate for 22 h in 2% (CI = −0.06 ± 0.02) and 10% (CI = 0.1 ± 0.1) FCS media. Then, a significant increase in CF migration was observed for 10% FCS in comparison with that for 0% and 2% FCS. The maximum value was reached at 72 h (0.72 ± 0.2) (*p* < 0.05). In turn, CF migration for 2% FCS (CI = 0.2 ± 0.003) was significantly higher compared to that for the control (CI = −0.004 ± 0.05) (*p* < 0.05) at 29 h of observation. This trend persisted until the end of the experiment.

rCFs did not migrate in the control medium. (Figure 6B). The rCF migration CI for 2% FCS was low. The highest migration rate was observed for 10% FCS, despite the low absolute CI value (CI = 0.2).

During the first 12 h, no difference between CF and rCF migration patterns were observed, which is associated with cell adhesion and their spreading at the plate’s bottom (Figure 6C). The CF migration CI from 24 h to the end of the experiment was significantly higher than that of rCFs for 10% FCS (Figure 6C), (*p* < 0.03). In the 2% FCS medium, the CF CI was higher than that of the rCFs starting from 36 h to the end of the experiment. In the control medium, CFs and rCFs did not migrate.

### 2.7. hCSC Protein Components Production

**Figure 7:**
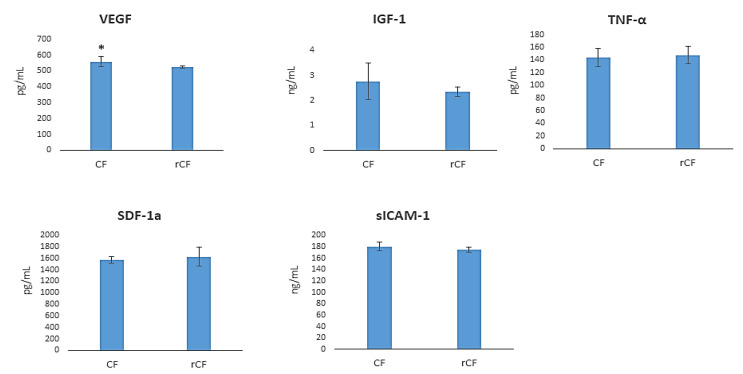
hCSC protein component production (*n* = 6, for each group). * *p* = 0.031.

The level of protein components was investigated in CF- and rCF-conditioned media by ELISA. While the CFs produced higher levels of VEGF relative to the rCFs (*p* = 0.031) (Figure 6), the levels of growth factors (SDF-1a, IGF-1), pro-inflammatory cytokine (TNF-α), and sICAM-1α were comparable in the media (Figure 7).

## 3. Discussion

Corneal keratocytes are quiescent cells, so it is difficult to expand them in vitro at a significant level for cell therapy and tissue engineering. This problem could be overcome by expanding CFs with high proliferative potential and their reversion into the keratocytes that maintain the morphofunctional properties of primary keratocytes. We have previously shown that ReLEx SMILE lenticules can be used to isolate the primary cultures of corneal fibroblasts [25]. In the present study, it has been demonstrated that fibroblasts from ReLEx SMILE lenticules reverse into keratocytes in serum-free KGM, maintaining the morphology and functional properties of primary keratocytes.

Previous studies demonstrated that CFs had the plasticity to differentiate into keratocytes [26,27]. This phenomenon was confirmed in our study where the CFs in KGM become similar to keratocytes. Here, we showed that fibroblast reversion was accompanied by a change in cell morphology. The rCFs acquired a dendritic shape and formed long cytoplasmic processes. They formed patterns shaped as waves and arcs (Figure 1B). The actin cytoskeleton stained with phalloidin demonstrated a sparse network of F-actin fibers, with a predominantly cortical actin organization (Figure 1D).

According to the TEM data, rCFs were more functionally active than CFs. This is confirmed by the high electron density of the cytoplasm, as well as a higher content of membranes of the rough endoplasmic reticulum and Golgi complex, and large activated nucleoli in the nucleus. The presence of large activated nucleoli in the rCF nucleus is a structural sign of the activation of synthetic processes. We suggest that fibroblast reversion leads to the activation of protein synthesis, in particular, keratocan, lumican, and extracellular matrix proteins. It is likely that the high mitochondrial content leads to an increase in rCF metabolism (Figure 2A,B). After reversion, rCFs had immunophenotypic signs of keratocytes. They expressed keratocyte markers such as keratocan, lumican, and ALDH1A1, and no markers of mesenchymal stem cells (CD90, CD73, and CD105) and adhesive molecules (Figure 3A,C).

Thus, cultivation in KGM reverts corneal fibroblasts into keratocytes and restores keratocyte markers (keratocan, lumican), except for CD34, being a marker of remaining keratocytes in vivo [28]. The role of CD34 expression in keratocytes has not been elucidated yet, but it is assumed that it is involved in the regulation of adhesion, differentiation, and quiescent states [28,29]. However, it has been shown that keratocytes express CD34 only in corneal stroma, and lose this marker in isolation [30].

It was previously reported that the cultivation of corneal fibroblasts in a medium with 10% FCS can result in their differentiation into myofibroblasts. [31]. Myofibroblasts are involved in corneal regeneration by causing wound contraction, fibrous extracellular matrix organization, and scar formation. These cells are characterized by a larger size compared to CFs, and a high level of α-SMA expression. The possibility of reversion of corneal myofibroblasts to fibroblasts and then to keratocytes, remains a controversial issue [32]. In our study, no changes were found in the morphology of corneal cells during their cultivation in a 10% FCS medium and no α-SMA expression in CF 3–6 passage was observed (Figure 3C). Therefore, the primary cells derived from lenticules in a medium containing 10% FCS were fibroblasts. This result agrees with the data of [27,33].

Fundebug et al. demonstrated that the primary keratocytes isolated from the corneal stroma caused a spontaneous formation of spheroids [34]. A similar phenomenon was observed in our study with rCFs. The spheroids increased in size by the eleventh day of cultivation, and the cells in the spheroids remained alive (Figure 4). The formation of spheroids is the indirect evidence that the keratocytes obtained from the fibroblast reversion acquire not only morphological, but also functional properties of primary corneal keratocytes.

Because keratocytes have low functional activity (do not proliferate or migrate in a healthy cornea), we investigated the functional activity of the rCFs in the presence of a mitogen (2% and 10% FCS). It was found that 10% FCS led to a significant increase in the CI proliferation of rCFs compared to the depleted medium (2% FCS) and control (0% FCS) (Figure 5). Thus, FCS is a powerful mitogenic stimulus to accelerate the proliferation of rCF. It should be noted that during the first four hours of the experiment, rCF CIs were comparable at 0%, 2%, and 10% FCS, probably due to cell adhesion. A slight activation of rCFs can be caused by a 0.25% trypsin/0.02% EDTA solution during the detachment of the adherent cells. Unlike corneal fibroblasts, rCFs after adhesion and spreading in a serum-free medium became dormant and do not proliferate until the end of the experiment. At the end of the experiment (after 120 h), fluorescent staining with acridine orange and propidium iodide was performed to show that the low proliferative activity of rCF was not associated with their apoptosis or death. Figure 5D shows that the rCFs were live (stained green with acridine orange).

We found that rCFs have low migratory activity compared to corneal fibroblasts (Figure 6). The growth factor concentration in FCS had a lesser effect on cell migration activity than on their proliferation. The rCF migration was weak even with the addition of 10% FCS. Thus, the reversion of fibroblasts into keratocytes is accompanied not only by changes in cell morphology, but also by changes in their functional activity (proliferation and migration). The low rCF proliferative and migratory potentials were similar to those of primary keratocytes.

The main function of primary keratocytes is associated with the synthesis and degradation of the extracellular matrix of the cornea. Therefore, it is important that rCFs support the synthesis of the extracellular matrix components. Healthy corneal keratocytes are characterized by a low expression of corneal extracellular matrix components (fibronectin and collagen) in contrast to CFs, and especially myofibroblasts [35,36]. Fibronectin and collagen play a key role in CF migration [36,37]. Figure 3C shows that rCFs expressed less fibronectin than CFs. We have previously shown that the production of fibronectin and total collagen by rCFs is comparable to that of CFs. Similarity in the production of fibronectin and collagen by rCFs is probably due to the initially low level of their production by CFs [25].

The cytokines and growth factors produced by cells in the cornea are involved in regulating chemotaxis, cell proliferation, migration, inflammation, normal angiogenesis, and wound healing [34]. We studied the effect of fibroblast reversion into keratocytes on the production of cytokines and growth factors. The protein components studied included VEGF, IGF-1, TNF-α, sICAM-1, and SDF-1a (Figure 7). These protein components maintain corneal homeostasis and are involved in inflammation. The analysis of biologically active substances in the culture medium showed that the rCFs produced less VEGF (*p* = 0.031) (Figure 7). VEGF production by many cells, including CFs, was shown in [38]. Philipp et al. demonstrated that, unlike activated fibroblasts, corneal stromal keratocytes weakly express VEGF [39]. The normal cornea is avascular, which causes its transparency. VEGF causes quiescent cells activation, so they participate in proliferation and migration regulation and form intercellular contacts [40]. The rCFs in our study did not proliferate and migrate, which can be associated with the low VEGF production in these cells.

We found no effect of the reversion of corneal fibroblasts into keratocytes on the production of IGF-1, TNF-α, sICAM-1, and SDF-1α. IGF-1, a protein of the insulin-like growth factor family, responsible for maintaining corneal homeostasis. This protein regulates the formation of a communication network (formation of a cell network) between keratocytes [41], proliferation, and differentiation of keratocytes into fibroblasts and myofibroblasts during inflammation and wounding [42]. Sarenak et al. showed that IGF-1 increases the secretion of keratocan, lumican, and cytosolic ALDH3A1. IGF-1 reduces the likelihood of scarring on the corneal stroma by increasing the proliferation of keratocytes and influencing wound healing [43]. IGF-1 secretion in corneal fibroblasts was shown by Ko et al. [44]. At the same time, Berthaut et al. found no IGF-1 expression in CFs [45]. In our study, we demonstrated IGF-1 production not only by rCFs, but also by fibroblasts.

The pro-inflammatory cytokine TNF-ά, adhesion molecules ICAM-1, and chemokine SDF-1 (stromal cell factor-1) play an important role in the regulation of allergic and inflammatory reactions during infectious and non-infectious processes in the cornea. These molecules ensure the migration of macrophages and leukocytes. They also promote tissue infiltration and the activation of polymorphonuclear neutrophils in the focus of inflammation [46,47,48]. Several cell types, including corneal fibroblasts, express TNF-ά [46] and SDF-1 [48]. The expression of the adhesion molecule ICAM-1 was shown on CFs [47] and keratocytes [49]. The similarity in the production of IGF-1, TNF-α, sICAM-1, and SDF-1a between rCFs and CFs indicates that the fibroblast reversion is not accompanied by induction of inflammation, and should be attributed to the healthy corneas used as a hCSC source.

Reversion CFs from ReLEx SMILE lenticules into keratocytes has been shown in the present study. The CF reversion into keratocytes was accompanied by changes in cell morphology and the expression of specific keratocyte markers (keratocan and lumican), as well as the loss of specific mesenchymal markers and their proliferative and migratory activities. The reversion of CFs into keratocytes was not accompanied by a change in the production of cytokines, with the exception of VEGF. rCFs were able to form spheres spontaneously. The study has demonstrated that keratocytes, derived through the reversion of fibroblasts from ReLEx SMILE lenticules, can be used for corneal tissue engineering and cell therapy of corneal pathologies.

The development of a new approach for the derivation of corneal stromal cells from the biological material usually disposed looks promising against the backdrop of a shortage of corneas throughout the world. The success of cell therapy depends not only on the choice of the cell source, but also on the microenvironment of the cells after their introduction. Corneal fibroblasts, being regional cells of mesenchymal origin, have the ability to produce extracellular matrix proteins. In addition, they are already committed to the cornea, so they can be more efficiently differentiated into keratocytes. Therefore, it is preferable to use corneal stromal cells for cell therapy and tissue engineering. In our opinion, this will ensure the formation of an adequate microenvironment and modulation of host keratocytes due to the paracrine effect of the cells when injected into the cornea.

## 4. Materials and Methods

### 4.1. Ethics Statement

The research involving humans was performed with the prior approval of the Local Ethics Committee of S. Fedorov Eye Microsurgery Federal State Institution (# 1 of 14 January 2021). All procedures were conducted in accordance with the principles and guidelines of the Declaration of Helsinki. Informed consent was obtained from all 30 myopic patients enrolled in this study in January–May 2021.

### 4.2. ReLEx SMILE

ReLEx SMILE surgeries were performed using the technology described by W. Sekundo [50]. The thickness of the upper tissue arcade (cap) was 120 µm, with an intended diameter of 7.5 mm, whereas the average diameter of the refractive lenticule varied from 6.0 to 7.0 mm. An VisuMaxTM FS laser system (Carl Zeiss Meditec, Jena, Germany) was used to perform a 90-degree lateral circumferential incision of 2.5 mm at the superior temporal position and a cap sidecut angle of 35–40 degrees.

Following the cutting procedure, the refractive lenticule was dissected, separated through the side-cut, and manually removed. A total of 60 stromal lenticules of 50–175 μm in thickness were obtained from 17 male and 13 female patients whose ages ranged from 18 to 37 years.

### 4.3. Corneal Stromal Cell Isolation and Expansion

Every two lenticules from each patient were cut with scissors and treated with 62.5 U/mL collagenase I (Sigma-Aldrich, Saint Louis, MO, USA) supplemented with 2% FCS (HyClone, FCS; Hyclone, Logan, UT, USA) at 37 °C for 18–20 h [17]. Then the tissue debris was removed by filtration through a 100 µm cell filter (BD Falcon, Brea, CA, USA). The cells were washed twice in a phosphate buffer solution (PBS), centrifuged at 402× *g* for 10 min and seeded onto a 24-well plate (TPP, Trasadingen, Switzerland). The keratocyte culture was obtained from these cells with cultivation in KGM containing advanced DMEM/F12, 10 ng/mL human basic fibroblast growth factor (bFGF; Sigma-Aldrich, Saint Louis, MO, USA), 1 mM L-ascorbate 2-phosphate (Sigma-Aldrich, Saint Louis, MO, USA), and ITS (insulin–transferrin–selenium solution, X100; Gibco, Carlsbad, CA, USA), 40 µg/mL gentamycin (Dalkhimpharm, Khabarovsk, Russia), 1% Gluta-MAX (Gibco, Carlsbad, CA, USA) at 37 °C in 5% CO_2_ conditions, with the culture medium replaced every 3–4 days. To obtain CFs, the extracted cells were cultured in DMEM/F12 supplemented with 10% fetal calf serum (FCS; Hyclone, Logan, UT, USA), 1% Gluta-MAX, 5 mM HEPES buffer (Sigma-Aldrich, Saint Louis, MO, USA), and 40 µg/mL gentamycin at 37 °C in 5% CO_2_ conditions until reaching a confluent monolayer, with the culture medium refreshed every 3–4 days. Once the primary cultures reached 80–90% confluence, the adherent cells were detached using a 0.25% trypsin/0.02% EDTA solution (Biolot, Saint Petersburg, Russia). CF passages 3 through 6 were used for further studies.

### 4.4. Corneal Fibroblast Reversion into Keratocytes

hCSCs were cultured in KGM during 21 days at 37 °C in 5% CO_2_ conditions, with the medium refreshed every 3–4 days. At the end of the differentiation, the cells were characterized and used in experiments.

### 4.5. Transmission Electron Microscopy

hCSCs were cultured in culture flasks, 25 cm^2^ (TPP, Trasadingen, Switzerland) at ~90% confluence, and then adhesive cells were detached from the plastic using a solution of 0.25% trypsin/0.02% EDTA (Biolot, Saint Petersburg, Russia). The cells were precipitated by centrifugation at 402× *g* for 10 min. Then the cell pellet was fixed with a 4% paraformaldehyde in the Hanks medium and 1% OsO4 solution (Sigma-Aldrich, Saint Louis, MO, USA) with phosphate buffer (pH 7.4) for 1 h, dehydrated in ethanol in ascending concentrations, and embedded in epon (Serva). Semithin 1 μ sections were prepared using the Leica EM UC7 microtome, stained with toluidine blue, and orientated for electron microscopy. The semithin sections with the thickness of 70–100 nm were contrasted with a saturated water solution of uranyl acetate and lead citrate, and analyzed using the JEM 1400 electron microscope (Jeol) (Multiple-access Center for Microscopy of Biological Subjects, Institute of Cytology and Genetics, Novosibirsk, Russia).

Morphometric measurements: hCSCs (50 cells per group) were analyzed at ×15,000 magnification using the ImageJ 1.48 v software (National Institutes of Health, Bethesda, MD, USA). The volume density of mitochondria, rough endoplasmic reticulum, Golgi complex, and lysosomes were counted.

Statistical analysis: the mean (M) and standard deviation (SD) were calculated using the Microsoft Excel 2016 software (Microsoft, Redmond, WA, USA). The significance of differences between the studied parameters was determined employing the Statistica 6.0 software (StatSoft, Tulsa, OK, USA), using the Mann–Whitney U-test at a confidence level of 95% (*p* < 0.05).

### 4.6. Phenotyping of Corneal Stromal Cells

The phenotype of obtained hCSCs was investigated in a CytoFLEX S flow cytometer (Beckman Coulter, Brea, CA, USA) using monoclonal antibodies to CD29 (100 µg/mL, cat. no. 303004), CD44 (50 µg/mL, cat. no. 338808), CD49a (200 µg/mL, cat. no. 328303), CD54 (100 µg/mL, cat. no. 353106) (all antibodies: Biolegend, San Diego, CA, USA), CD34 (25 µg/mL, cat. no. 345801), CD45 (6 mg/mL, cat. no. 332784), CD184 (20 µL per test, cat. no. 555976), HLA DR (1:20, cat. no. 560652), CD73 (6.3 µg/mL, cat. no. 550257), CD90 (0.5 mg/mL, cat. no. 561969), and CD105 (100 µg/mL, cat. no. 562408) (all antibodies: BD Biosciences Pharmingen, San Diego, CA, USA), and monoclonal antibodies to keratocan (1:50, cat no. orb7921; Biorbyt, Cambridge, UK), lumican (1:50, cat no. orb124743; Biorbyt, Cambridge, UK), and aldehyde dehydrogenase 1 family member A1 (ALDH1A1) (1:50, cat no. 32005-05161, AssayPro, St. Charles, MO, USA), following the instructions provided by the manufacturer. Briefly, the culture medium was removed, the culture flasks were washed once with PBS, and the adherent cells were detached using a 0.25% trypsin/0.02% EDTA solution (Biolot, Saint Petersburg, Russia). Then the cells were harvested, washed, and the cell number adjusted to a concentration of 1 × 10^6^ cells/mL in ice-cold FACS Buffer (PBS, 1% BSA, 0.1% NaN_3_ sodium azide). The cells were stained in BD Falcon tubes in 100 μL cell suspension and 0.1–10 μg/mL antibody added to each tube and incubated for 30 min at 4 °C in the dark. Then, the cells were centrifuged 3× *g* times in 1 mL of BSA-free ice-cold FACS buffer at 402× *g* for 5 min. The pellet was suspended in 400 µL of FACS buffer. To analyze keratocan, lumican, and ALDH1A1 expression, the cells were harvested, washed with PBS, fixed and permeabilized using 0.2% Tween20 (Merck, Millipore, Sigma-Aldrich, Supelco, Germany), following the instructions provided by the manufacturer. Then the cells were stained with rabbit anti-human keratocan, rabbit anti-human lumican or rabbit anti-human ALDH1A1 antibody (1:50), and washed three times with PBS. The stained cells were analyzed using the flow cytometer, a minimum of 10,000 events per experiment.

### 4.7. Immunocytochemistry

Cells of 3–6th passage were attached to L-polylysine-coated glass slides, fixed in 4% paraformaldehyde, permeabilized with 0.1% Triton X-100 (Bio-Rad, Hercules, CA, USA) for 20 min at room temperature, and then blocked with 1% bovine serum albumin (Sigma-Aldrich, St. Louis, MO, USA) at room temperature for 1 h, and incubated with mouse anti-CD90 (1:100, cat. no. ab134358; Abcam, Cambridge, UK), rabbit anti-alpha smooth muscle actin (1:100, cat. no. A17910; ABclonal, Wuhan, China), mouse anti-fibronectin (1:100, cat. no. ab6328; Abcam, Cambridge, UK) monoclonal antibody conjugated with fluorochrome for 12 h at 4 °C. Then, the cells were incubated for 40 min at room temperature with secondary antibodies (goat anti-mouse IgG conjugated with Alexa Fluor 488, 1:400, cat. no. A11029; Thermo Fisher Scientific, Waltham, MA, USA and goat anti-rabbit IgG conjugated with Alexa Fluor 488, 1:400, cat. no. ab150077; Abcam, Boston, MA, USA) and washed. The cell nuclei were stained with 4′,6-diamidino-2-phenylindole (DAPI, Abcam, Cambridge, UK). The actin–cytoskeleton of the cells was stained with phalloidin coupled with Alexa Flour 488 (1:200, cat. no. A12379, Thermo Fisher Scientific, Waltham, MA, USA) for 60 minutes, as per the manufacturer’s instructions. The cells were photographed using an Axio Observer microscope (Carl Zeiss, Oberkochen, Germany).

### 4.8. Sphere Formation of Reversed Fibroblasts

In the case of the fibroblasts being reversed into the keratocytes that spontaneously formed into spheres, the last were characterized (their diameter, area, and number) using an Axio Observer microscope (Carl Zeiss, Oberkochen, Germany).

### 4.9. Real-Time Cell Proliferation Monitoring

An xCELLigence impedance-based real-time cell analyzer (RTCA) (Roche, Applies Science, Indianapolis, IN, USA) was used to assess CF and rCF proliferation. The cells were seeded at densities of 1 × 10^4^ cells/well into an E-plate 16 (ACEA Biosciences, San Diego, CA, USA) with 200 μL media containing 2% FCS or 10% FCS, and cultivated at 37 °C in a humidified 5% CO_2_ incubator. The impedance value of each well was monitored automatically by the RTCA every hour for 120 h. The electrical impedance was measured by the RTCA and quantified by its software v. 1.2.1 as a dimensionless parameter, cell index (CI), which allowed for the analysis of cell adhesion, spreading, proliferation, cell viability/death, and detachment in real time.

### 4.10. Real-Time Cell Migration Monitoring

Real-time cell migration was measured using the xCELLigence RTCA DP instrument equipped with a CIM-plate 16. The plate is a 16-well system, where in each well, the upper and lower chambers are separated by an 8 μm microporous membrane. CFs and rCFs were seeded at densities of 1 × 10^4^ cells/well into the upper chamber in a serum-free medium. DMEM/F12 and KGM supplemented with 2% and 10% FCS were placed in the bottom chambers as a chemoattractant. The migration was measured as the relative rate of change (CI) across the microelectronics integrated into the bottom side of the membrane. The impedance value of each well was monitored automatically by the instrument every hour for 72 h. The result was quantified using the RTCA software v. 1.2.1.

### 4.11. Obtaining Conditioned Media

After the culture reached 90–100% confluence, the medium was removed and the culture flasks were washed thoroughly with PBS, and a fresh growth medium without FCS was added. The cells were cultured for 24 h and then the culture supernatants were collected and stored at −20 °C.

### 4.12. ELISA

Commercially available ELISA kits were used to determine the human insulin growth factor-1 (IGF-1), human free brain-derived neurotrophic factor (BDNF) (all kits: R&D Systems, Minnesota, MN, USA), human stromal-derived factor-1a (SDF-1a; Thermo Scientific, Waltham, MA, USA), human vimentin, total collagen, human fibronectin (all kits: Abcam, Boston, MA, USA), human soluble intercellular adhesion molecule-1 (sICAM-1; Affymetrix eBioscience, Santa Clara, CA, USA), vascular endothelial growth factor (VEGF), erythropoietin (EPO), and tumor necrosis factor-alpha (TNF-α) (all kits: Vector-BEST, Novosibirsk, Russia), produced by the CFs and rCFs. The kits were applied as per the manufacturer’s recommendations.

### 4.13. Statistical Analysis

Statistical analysis was performed using Statistica 10.0 (Stat Soft Inc., Tulsa, OK, USA). The Shapiro–Wilk test was used to confirm the data distribution normality. The obtained data were presented as the mean ± SD (standard deviation) and analyzed by a one-way analysis of variance (ANOVA), followed by Bonferroni’s multiple comparison post hoc test. Statistical significance between the groups was established for *p* < 0.05.

## 5. Conclusions

The present study showed that fibroblasts isolated and expanded from ReLEx SMILE lenticules can be a source of keratocytes. Fibroblast reversion was accompanied by a change in cell morphology. rCFs had a dendritic shape with long cytoplasmic processes, and formed patterns shaped as waves and arcs. The ultrastructure of the rCFs was characterized by a high electron density of the cytoplasm and a high content of organelles, which indicates the activation of synthetic processes within them. The reversed keratocytes expressed specific markers of keratocytes (keratocan and lumican) and had the ability to spontaneously form spheroids. rCFs had low functional activity (proliferated and migrated only in the presence of the mitogen, FCS), and their conditioned medium contained low levels of VEGF.

Keratocytes derived through the reversion of fibroblasts from ReLEx SMILE lenticules can be used for tissue engineering and cell therapy of various corneal pathologies associated with the loss of stromal cells.

## Data Availability

Not applicable.
